# Supplementary material for: A route to engineered high aspect-ratio silicon nanostructures through regenerative secondary mask lithography
Source: Nanoscale. 2022 Jan 18;14(5):1847–54. doi: 10.1039/d1nr07024j (PMC9115640; doi:10.1039/d1nr07024j)
Supplement: NR-014-D1NR07024J-s001 [file NR-014-D1NR07024J-s001.pdf]

Electronic Supplementary Material (ESI) for Nanoscale.
This journal is © The Royal Society of Chemistry 2022

Supporting Information

A route to engineered high aspect-ratio silicon nanostructures through regenerative secondary mask lithography

Martyna Michalska,†[a] Sophia K. Laney,†[a] Tao Li,†[a] Manish K. Tiwari,[b,c] Ivan P. Parkin[d] and Ioannis Papakonstantinou[a]*

[a] *Photonic Innovations Lab, Department of Electronic & Electrical Engineering, University College London, Torrington Place, London WC1E 7JE, UK. i.papakonstantinou@ucl.ac.uk*

[b] *Nanoengineered Systems Laboratory, Department of Mechanical Engineering, University College London, Torrington Place, London WC1E 7JE, UK.*

[c] *Wellcome/EPSRC Centre for Interventional and Surgical Sciences (WEISS), University College London, London, W1W 7TS, UK.*

[d] *Department of Chemistry, University College London, Torrington Place, London WC1E 7JE, UK.*

† These authors contributed equally to this work.

Supporting Figures

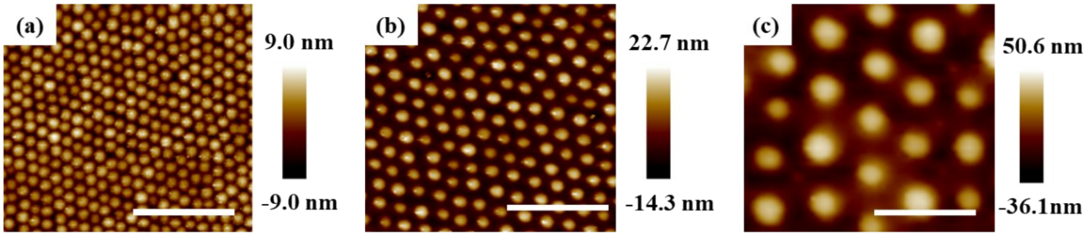

Figure S1. AFM images of hexagonally packed PS-*b*-P2VP micellar bumps generated from direct spin coating onto Si/SiO_2. The molecular weight M_w dictates the pitch p of the micelles and varies across (a-c) with M_w / kg mol^{-1} = 57-*b*-57, 109-*b*-90, and 440-*b*-353, giving rise to p = 56, 95, and 257 nm, respectively. Scale bars = 500 nm.

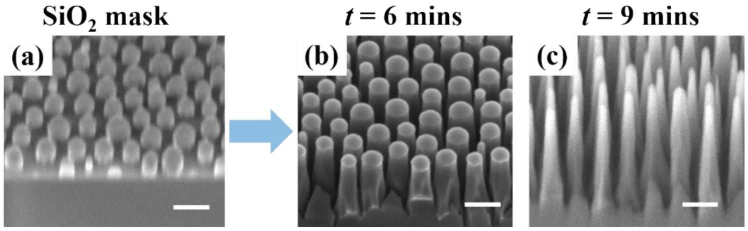

Figure S2. SEM images of the structures generated from a hard mask with d = 112 nm. (a) SEM image of the hard mask, which under coil power 300 W and platen power 40 W yields straight walled nanopillars (b) with mask remaining after 6 min. Etching for a further 3 min under the same condition yields slender high AR nanopillars. Scale bars = 200 nm.

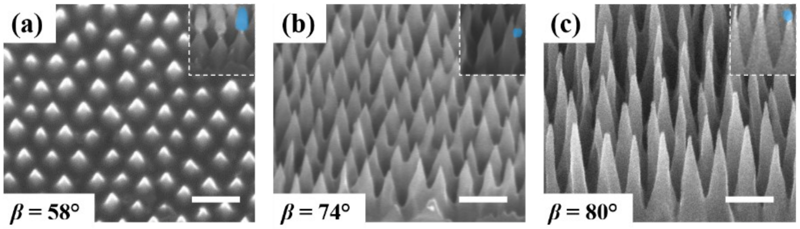

Figure S3. SEM images of sharp nanocones with varying slope angles after HF treatment (inset = before HF treatment, with remaining SiO_2 hard mask highlighted in blue). (a) Nanocones with β = 58°, generated from a hard mask of h = 70 nm under mixed plasma of Cl_2 (18 sccm) and SF_6 (2 sccm) at coil power 300 W, and platen power 10 W for 3 min. (b) Nanocones with β = 74°, generated from a hard mask of h = 40 nm under Cl_2 plasma (20 sccm) at coil power 200 W, and platen power 10 W for 25 min, followed by 1 min etching under mixed plasma: Cl_2 (19 sccm) and SF_6 (1 sccm). (c) Nanocones with β = 80°, generated from a hard mask of h = 100 nm under Cl_2 plasma (20 sccm) at coil power 200 W, and platen power 15 W for 15 min. Scale bars = 200 nm.

Supporting Videos

Supporting Video 1 – Slow-motion video of a bouncing droplet upon impacting the nanostructured 6-inch silicon wafer. The video was recorded using a high-speed camera. A water droplet of 8μL was deposited from a height of 1 cm, giving rise to 19 bounces.

Supporting Video 2 – Self-cleaning effect of the superhydrophobic and antireflective 6-inch silicon wafer. The self-cleaning effect enables efficient removal of black pepper powder by a stream of water.
